# Novel Insights into the Acute and Sublethal Toxicity of Layered Double Hydroxide Nanoparticles in *Spodoptera frugiperda* Larvae

**DOI:** 10.1021/acsomega.6c06039

**Published:** 2026-09-23

**Authors:** André Lopes Ferreira, Kelly Cristina Gonçalves, Sofia Cristina Picotte, Raqueli Viana, Ricardo Antonio Polanczyk, Marcelo Bispo de Jesus

**Affiliations:** † NanoCell Interactions Lab., Departamento de Bioquímica e Biologia Tecidual, Instituto de Biologia, Universidade Estadual de Campinas - UNICAMP, Campinas, São Paulo 13083-872, Brazil; ‡ Department of Plant Protection, 153996 Universidade Estadual Paulista Julio de Mesquita Filho - UNESP, São Paulo, São Paulo 14884-900, Brazil

## Abstract

Nanotechnology is increasingly integrated into agriculture, yet some nanomaterials are investigated primarily as delivery platforms, nanofertilizers, controlled-release pesticides, antimicrobials, or oligonucleotide delivery systems, while their intrinsic biological activity remains largely unexplored. Layered double hydroxide (LDH) transition metal containing are widely assumed to be biologically inert despite their tunable metal composition, high surface reactivity, and potential to interact directly with biological systems. Here, we evaluated the acute and sublethal toxicity of LDH nanoparticles containing transition metals (Al–Ni, Al–Co, or Al–Cu) on *Spodoptera frugiperda* larvae. Laboratory bioassays were performed by incorporating nanoparticles into the larval artificial diet to quantify dose–mortality relationships, estimate LC_50_ values, and assess impacts on larval and pupal development. All LDH formulations exhibited dose-dependent toxicity, with Al–Cu LDH showing the highest potency and the lowest LC_50_ (19.1–37.3 mg mL^–1^). Furthermore, a significant reduction in pupation rate and larval weight was observed, suggesting relevant sublethal effects. These findings provide evidence that metallic LDHs possess bioactivity against an agriculturally important pest, challenging the assumption of LDH inertness and revealing their potential as dual-function materials that combine inherent biological effects with delivery capabilities. This work establishes a new foundation for evaluating LDHs within pest-management frameworks and underscores the need to assess selectivity, environmental fate, and impacts on nontarget organisms before field deployment.

## Graphical abstract

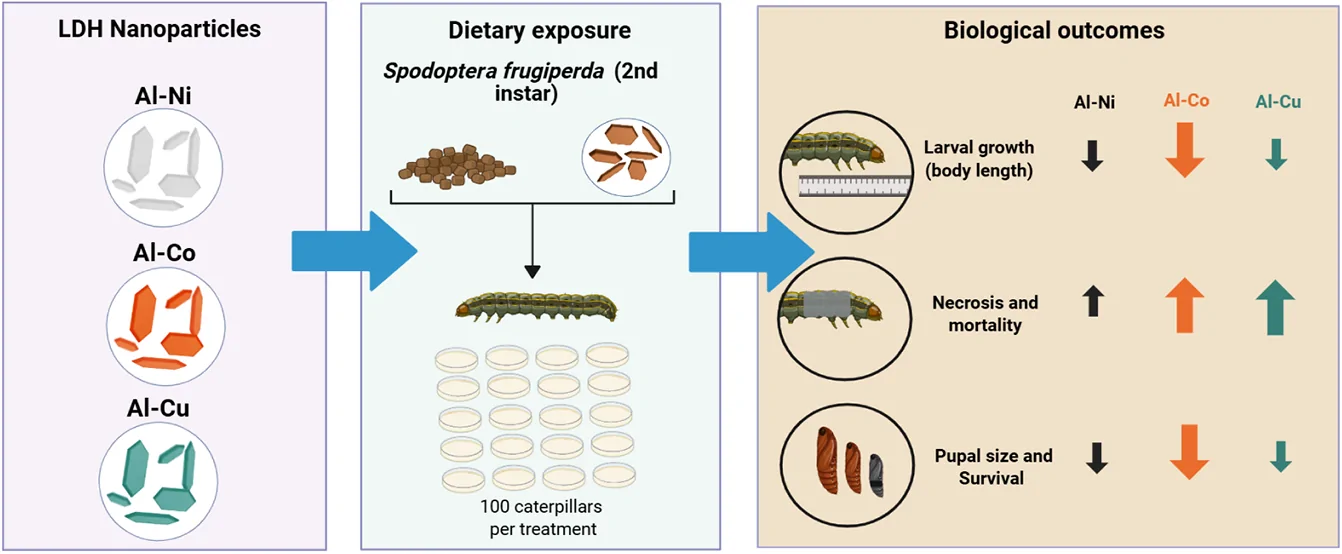

## Introduction

The increase in the world population continues to intensify the demand for food, reinforcing the need for agricultural practices that ensure both food security and production sustainability. However, crop yields are constrained by multiple factors, including abiotic stresses such as drought, low temperatures, salinity, and soil waterlogging. Biotic pressures exerted by pathogenic microorganisms (bacteria, viruses, fungi, and parasites), weeds, and insect pests further exacerbate these limitations.^1^ These environmental and biological stressors trigger complex physiological responses in plants, compromising their growth, metabolism, and development. Among these biotic stressors, insect pests constitute one of the most pervasive and economically damaging threats to global agricultural productivity.

Arthropods account for substantial annual losses, estimated at 18–20% of total production, which represents an economic impact of approximately USD 470 billion,^2^ driving extensive reliance on chemical control strategies. Among these pests, the fall armyworm (*Spodoptera frugiperda*) stands out as one of the most destructive agricultural pests due to its exceptional polyphagy, capable of feeding on over 350 plant species, including major crops such as maize, rice, wheat, and sorghum.^5^ Moreover, *S. frugiperda* exhibits rapid development, high dispersal capacity, and a remarkable capacity for resistance evolution. *S. frugiperda* is particularly devastating in tropical and subtropical regions; for example, annual losses exceed USD 9 billion across sub-Saharan Africa and around USD 400 million in Brazil because under severe infestation, maize productivity can decrease by 50% to 80%.^3–5^ Given its biology and widespread resistance to conventional insecticides, *S. frugiperda* has become a central driver of pesticide use and an urgent priority for the development of more efficient, selective, and sustainable technologies.

In contemporary agriculture, the use of agrochemicals, including fertilizers and pesticides remains critical for ensuring crop productivity, although their intensive and recurrent use has raised serious concerns. Residues in food, soil contamination, and water pollution pose risks to human and animal health, underscoring the need for safer and more environmentally responsible alternatives.^6,7^ Many pest species exhibit high reproductive rates, remarkable adaptive capacity, and develop resistance to pesticides, progressively reducing the effectiveness of current management strategies.^8,9^ Moreover, the recurrent use of broad-spectrum insecticides can disrupt ecological balance, harm beneficial organisms, and promote pest resurgence. Climatic conditions also play a crucial role, as variations in temperature and humidity directly influence pest distribution, reproduction, survival, and infestation intensity. In light of these multiple obstacles, it becomes evident that innovative, selective, and sustainable approaches are essential to achieving more efficient and resilient agricultural pest management.^10^

In response to these challenges, nanomaterials have emerged as a promising class of tools capable of enabling more selective, efficient, and sustainable pest-management strategies.^1^ Among engineered nanomaterials^11^ Layered Double Hydroxides (LDHs) containing transition metal stand out due to their positively charged surfaces, tunable metal composition, and high anion-exchange capacity, properties that strongly influence their interactions with biological systems.^12,13^ These attributes position LDHs as a compelling candidate for agricultural delivery platforms, yet their intrinsic biological activity has rarely been investigated, and LDHs are still broadly assumed to be biologically inert.

We therefore hypothesized that LDHs, particularly those containing transition metals, could induce detectable acute or developmental effects in *S. frugiperda*. Testing this hypothesis would not only clarify the intrinsic biological activity of these materials but also determine whether LDHs could serve as inherently bioactive platforms for future delivery strategies in pest management. To test this hypothesis, we followed a logical experimental framework that integrates (*i.*) acute toxicity assays for determination of dose–mortality relationships and LC_50_ values, (*ii*.) sublethal developmental assessments to quantify impacts on larval growth, pupal formation, and metamorphosis, and, (*iii.*) comparative analysis among LDH compositions (Al–Ni, Al–Co, Al–Cu) to understand how metal composition influences acute and sublethal responses. By combining these complementary approaches, this study aims to determine not only whether LDHs can affect survival and development, but also whether their effects are sufficient to justify consideration of LDHs as stable, biocompatible, and potentially materials for pest management.

## Materials and Methods

### Synthesis and Characterization of Nanoparticles

Layered double hydroxides (LDHs) were synthesized by the coprecipitation method. Saline solutions (10 mL) containing M2+ (3.0 mmol L^–1^) and M3+ (1.0 mmol L^–1^) were slowly added to 40 mL of NaOH (0.15 mol L-1), previously deaerated with N2, under vigorous stirring for 20 min in an inert atmosphere. The resulting precipitate was separated by centrifugation, washed, and subjected to hydrothermal treatment at 100 °C for 16 h. The formed crystals were redispersed in 50 mL of deionized water, yielding a homogeneous, stable suspension. Dynamic light scattering (DLS) and zeta potential analyses were performed using a ZetaSizer Nano ZS 90 (Malvern, UK) at 25 °C and a 90° angle, using standardized suspensions at 0.1 mg mL^–1^. A more comprehensive physicochemical characterization of these formulations (including transmission electron microscopy (TEM), X-ray diffraction (XRD), Fourier-transform infrared spectroscopy (FTIR), and long-term colloidal stability analyses) has been reported in our complementary study.^14^

### Rearing of *S. frugiperda*

The colony was maintained at the Laboratory of Microbial Control of Pest Arthropods, FCAV/UNESP. Adults were kept in plastic cages (35 cm × 30 cm) and fed with a 10% honey solution. Larvae were fed an artificial diet composed of common bean (*Phaseolus vulgaris*), wheat germ, and soy protein,^15^ prepared in the laboratory. Eggs were collected daily. After hatching, larvae were placed individually in plastic containers (7 cm diameter; 3 cm height) and fed the artificial diet until pupation. Pupae were sexed and transferred to cages until adult emergence. Insects were reared in a climate-controlled chamber at 25 ± 2 °C, 60–70% relative humidity, and a 16:8 h (light:dark) photoperiod.

### Lethal Concentration (LC_50_) Bioassays

Preliminary screening assays were conducted to determine the range of concentrations to be used in the experiments. The selected concentrations were Al–Ni (8–100 mg mL^–1^), Al–Co (3–100 mg mL^–1^), Al–Cu (1–100 mg mL^–1^), and an untreated control. Each treatment consisted of in 100 insects per treatment.

### Mortality Bioassays

Nanoparticles were diluted in sterile deionized water, and 80 μL of the suspension was uniformly applied to the surface of the diet contained in transparent cylindrical plastic containers (Ø = 2.5 cm; volume = 1.5 cm^3^). Sterile deionized water was used as the control. After application, the diets were allowed to dry completely, and one second-instar larva was then placed in each container (n = 100).

### Sublethal Effects on *S. frugiperda* Larval Development

To evaluate the effects of each nanoparticle treatment on larval development, surviving larvae were weighed from day 12 onward, at two-day intervals, using an analytical balance, until pupation, when the pupae were also weighed. The measurements of the larvae and pupae were then compared with the control group to identify possible developmental differences.

### Statistical Analysis

Statistical analyses were performed using GraphPad Prism version 8.0.2 (GraphPad Software, San Diego, CA, USA). Mortality data were analyzed by nonlinear regression using a four-parameter logistic model after logarithmic transformation of the concentrations to estimate the median lethal concentration (LC_50_) and the corresponding 95% confidence intervals. Larval and pupal weight data are presented as mean ± standard deviation (SD). Differences among treatments were analyzed independently at each assessment point using one-way analysis of variance (ANOVA), followed by a multiple-comparison test. Pupation rates were expressed as percentages and analyzed using one-way analysis of variance (ANOVA) followed by a multiple-comparison test. Statistical significance was established at *p* < 0.05.

## Results and Discussion

### Acute Toxicity and Concentration-Dependent Mortality

Understanding the direct biological effects of LDH nanoparticles on *Spodoptera frugiperda* is central to evaluating their potential as novel pest-management tools. *S. frugiperda* is an exceptionally damaging and resilient lepidopteran pest, with well-documented resistance to multiple classes of conventional insecticides and Bt crops, which makes the discovery of new modes of action particularly valuable.^3,4,16^ All three formulations were previously confirmed to exhibit the characteristic lamellar structure, hexagonal platelet morphology, and FTIR spectral signatures of LDH materials.^14^ The hydrodynamic diameters and zeta potentials measured in the present study are comparable to those reported in the complementary study within expected batch-to-batch variability: 266.8 nm and +41.49 mV (Al–Ni), 223.8 nm and +47.0 mV (Al–Co), and 241.3 nm and +46.0 mV (Al–Cu). Given this context, assessing mortality following exposure to metallic LDHs provides critical insight into whether these nanomaterials can exert biologically meaningful pressure on a species that is notoriously difficult to suppress. The use of *S. frugiperda*, a species that is widely distributed, agriculturally significant, and easily maintained under laboratory conditions, enables controlled and reproducible evaluation of these potential effects. The results obtained from the acute toxicity assays (Figure 1) demonstrated that Layered Double Hydroxide (LDH) nanoparticles containing transition metals (Al–Ni, Al–Co, and Al–Cu) caused larval mortality in *S. frugiperda* in a clearly concentration-dependent manner.

**Figure 1:**
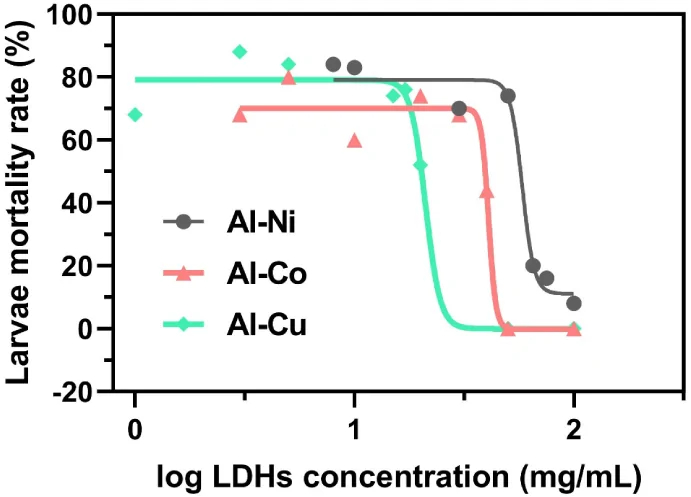
Acute toxicity assays and LC_50_ estimates for *Spodoptera frugiperda* larvae exposed to Layered Double Hydroxide (LDH) nanoparticles containing transition metals. Nonlinear regression curves fitted to the same data (logarithmic transformation of concentrations) used for LC_50_ calculation, indicating the following ranges: Al–Ni = 53.7–64.9 mg mL^–1^, Al–Co = 36.8–46.3 mg mL^–1^, and Al–Cu = 19.1–37.3 mg mL^–1^.

Al–Ni LDH nanomaterial exhibited progressive mortality with increasing concentration, reaching 92% at 100 mg mL^–1^, with an LC_50_ of 58.12 mg mL^–1^ (53.7 to 64.9 mg mL^–1^; dark gray line in Figure 1). The ingestion of nickel nanoparticles may result in their accumulation in the gastrointestinal tract,^17^ causing histological alterations, physiological dysfunctions, and imbalance of the gut microbiota.^18^ Furthermore, internalized particles can induce cellular and tissue damage either through direct contact with organelles or through the release of Ni^2+^ ions following dissolution, acting as an intracellular nickel delivery system capable of causing ionoregulatory, oxidative, or respiratory stress associated with dissolved Ni toxicity.^19,20^

Al–Co nanoparticles exhibited a marked increase in mortality even at lower concentrations, with 40% mortality observed at 10 mg mL^–1^, reaching 100% mortality at 50 mg mL^–1^. The estimated LC_50_ was 40.81 mg mL^–1^ (36.8–46.3 mg mL^–1^; red line in Figure 1), indicating higher relative toxicity compared to the nickel-based material. One hypothesis for this difference may be related to the higher redox potential of cobalt, which can induce oxidative stress through the generation of reactive oxygen species (ROS), affecting larval metabolism and compromising epithelial tissues.^21,22^

Al–Cu nanoparticle formulation provided the lowest LC_50_ of 21.05 mg mL^–1^ (95% CI: 19.1 to 37.3 mg mL^–1^), indicating greater toxicity even at lower concentrations (green line in Figure 1) when compared with the other nanoparticle formulations (Al–Ni and Al–Co). Despite the nonlinear initial response observed at lower doses, mortality increased sharply above 20 mg mL^–1^. One possible explanation for the elevated toxicity can be attributed to the partial degradation of nanoparticles in the larval intestinal tract and the consequent release of Cu^2+^ ions, known to interact with essential proteins and enzymes, leading to denaturation and homeostatic imbalance in the larvae. Moreover, metallic nanoparticles can inhibit substrate migration across cell membranes, disrupt basic metabolic processes in electron transfer systems, and impair respiration in insect pests.^23,24^ Although our study did not aim to fully elucidate the mechanisms triggered by nanoparticle exposure, the literature provides some insights, for instance, NPs can easily bind to and disrupt biological molecules such as DNA, lipids, and proteins, potentially causing histological damage and malformations.^18,25^

Taken together, the results demonstrate that all tested LDH formulations exert dose-dependent toxicity against *S. frugiperda*, with copper-containing LDHs producing the most pronounced effects, followed by cobalt and nickel. By quantifying mortality after 10 days of exposure, this study provides evidence that LDH nanoparticles can directly compromise the survival of a major agricultural pest, positioning metallic LDHs as candidates for futures studies on insect-control technologies. The concentration range evaluated in this study was selected to establish dose–response relationships and determine LC_50_ values under controlled laboratory conditions. Therefore, the tested concentrations should be interpreted as part of an initial toxicological screening rather than a direct simulation of agricultural application rates. Although the highest concentrations may exceed those expected under field conditions, they provide important information regarding the biological activity and toxicological profile of transition-metal-containing LDHs.

### Evaluation of Sublethal Effects

Acute mortality provides an essential indication of the biological activity of LDH nanoparticles against *S. frugiperda*, yet effective pest-management technologies also depend on their capacity to disrupt key physiological processes. Because growth suppression and delayed development are critical determinants of population dynamics in this resilient pest, we evaluated whether LDHs impose additional sublethal effects beyond lethality. Assessing changes in larval body mass thus offers a sensitive measure of metabolic and energetic disruption, further revealing the extent to which these nanomaterials can compromise the performance and fitness of *S. frugiperda*. Exposure of *S. frugiperda* larvae to different compositions of Layered Double Hydroxides (Al–Ni, Al–Co, and Al–Cu) produced distinct patterns of sublethal growth inhibition that varied with nanoparticle composition, concentration, and time of exposure (Figure 2A–D).

**Figure 2:**
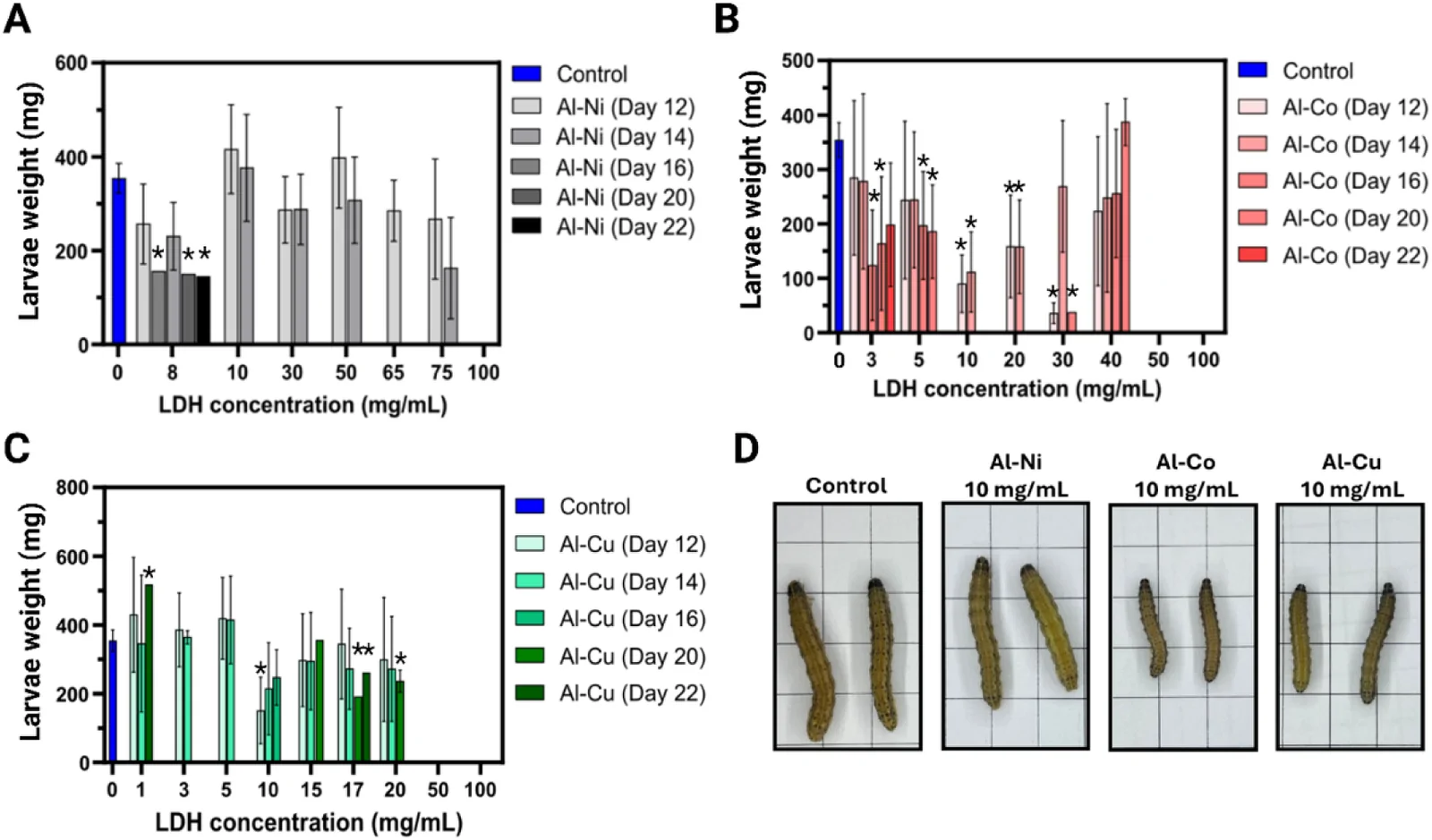
Effect of different compositions of layered double hydroxides (LDHs) on the larval growth of *Spodoptera frugiperda*. Mean weight of larvae fed with diets containing different concentrations of Al–Ni (A), Al–Co (B), and Al–Cu (C) nanomaterials over 22 days of exposure. A significant reduction in larval weight was observed at concentrations above 8 mg mL^–1^ for Al–Ni, 5 mg mL^–1^ for Al–Co, and 3 mg mL^–1^ for Al–Cu, indicating a dose-dependent effect and marked toxicity of systems containing transition metals. (D) Representative images of larvae exposed to a concentration of 10 mg mL^–1^ show a visible reduction in body size compared to the control, corroborating the quantitative results. Photographs were taken using a reference grid with approximately 1 cm spacing to facilitate visualization of differences in body size and developmental delay. Differences among treatments were analyzed independently at each assessment time point using one-way ANOVA followed multiple comparison test (*p* < 0.05). Asterisks (*) indicate significant differences compared to the control group. Photographs were taken by André Lopes Ferreira.

Al–Ni LDHs (Figure 2A) led to a marked reduction in larval mean weight, particularly at 8 mg mL^–1^, where the average weight decreased from approximately 354 mg in the control to values close to 150–144 mg on days 20 and 22 of evaluation. This inhibitory effect on larval growth was significant (*p* < 0.05) and persisted over time; which could be attributed to the gradual release of Ni^2+^ from the matrix interfering with essential metabolic processes, reducing nutrient assimilation and biomass accumulation. Higher concentrations (10–75 mg mL^–1^) did not further enhance the inhibitory effect during the initial weighing days; however, concentrations above 10 mg mL^–1^ ultimately resulted in complete larval mortality, indicating that the growth effects observed at lower concentrations represent an early manifestation of toxicity.

Al–Co LDHs (Figure 2B) exhibited the most sublethal effects among the tested formulations. Significant reductions in larval weight were observed from 3 mg mL^–1^ (*p* < 0.05), with mean values frequently below 200 mg even in the early days of exposure. This trend persisted at intermediate concentrations (5–30 mg mL^–1^), indicating that small amounts of Al–Co LDH are sufficient to induce severe physiological stress. Cobalt is known to participate in redox reactions capable of generating reactive oxygen species (ROS), leading to lipid peroxidation and mitochondrial damage.^26,27^ The resulting metabolic disruption likely impairs energy allocation and growth, contributing to the rapid decline in body mass.

Al–Cu LDHs (Figure 2C) displayed a nonlinear response. At low concentrations (3 mg mL^–1^), a significant increase in body weight was observed compared to the control (*p* < 0.05), reaching mean values close to 500 mg. However, higher concentrations (10–20 mg mL^–1^) resulted in a significant mass reduction, with values ranging between 200 and 300 mg. This reversal in response is consistent with the dose-dependent toxicity of Cu^2+^, potentially reflecting increased ionic release at higher concentrations and the resulting disruption of cellular homeostasis.

Although Al–Cu LDH exhibited the highest acute toxicity, reflected by lower LC_50_ values and increased mortality, Al–Co LDH frequently produced more consistent sublethal effects on larval growth and development. These findings suggest that acute toxicity and developmental impairment may not necessarily follow the same toxicity pattern among transition metal containing LDHs indicating that different formulations may affect distinct physiological processes.

The reductions in larval mass were consistent with the morphological alterations observed in Figure 2D. Larvae exposed to 10 mg mL^–1^ of any LDH formulation showed reduced body size and darker pigmentation compared to the control, changes commonly associated with physiological stress, impaired development, and metal accumulation.^28–30^ These morphological effects indicate not only growth retardation, but also physiological alterations associated with intoxication processes and, possibly, metal accumulation. Overall, the results suggest that LDHs containing transition metals (Ni, Co, and Cu) distinctly modulate the larval growth of *S. frugiperda*. Among them, Al–Co exhibited the highest inhibitory potential, followed by Al–Ni and Al–Cu. The pronounced weight suppression suggests that multiple toxicity pathways may be triggered, including metal-ion release and the affinity of each metal for biomolecules in the digestive tract. Taken together, these findings demonstrate that LDH nanoparticles exert not only acute toxicity but also pronounced sublethal impairments on larval development, strengthening the case for their evaluation as innovative pest-control materials.

### Evaluation of Sublethal Effects on Pupal Weight and Pupation Rate

Building upon the pronounced effects of LDH nanoparticles on larval growth, we next evaluated whether these materials also disrupt later developmental transitions in *S. frugiperda*. For formulations containing Al–Ni (Figure 3A), the mean pupal weight ranged from approximately 210 to 240 mg at concentrations between 8 and 75 mg mL^–1^, values close to the control (211 ± 20 mg). This indicates that individuals able to complete metamorphosis did not exhibit severe impairment in pupal development; however, the pupation rate was severely impaired. The control group exhibited about 75% pupation, whereas larvae exposed to 8 mg mL^–1^ of Al–Ni showed a pupation rate close to 12%, representing an 84% reduction, and at higher concentrations, the rate remained below 30% (Figure 3D). These findings imply that Al–Ni primarily disrupt the larval-to-pupal transition, possibly through metabolic or hormonal disturbances that affected the larvae development, rather than affecting the morphology of pupae that failed to develop successfully, as previously reported in the literature.^31^

**Figure 3:**
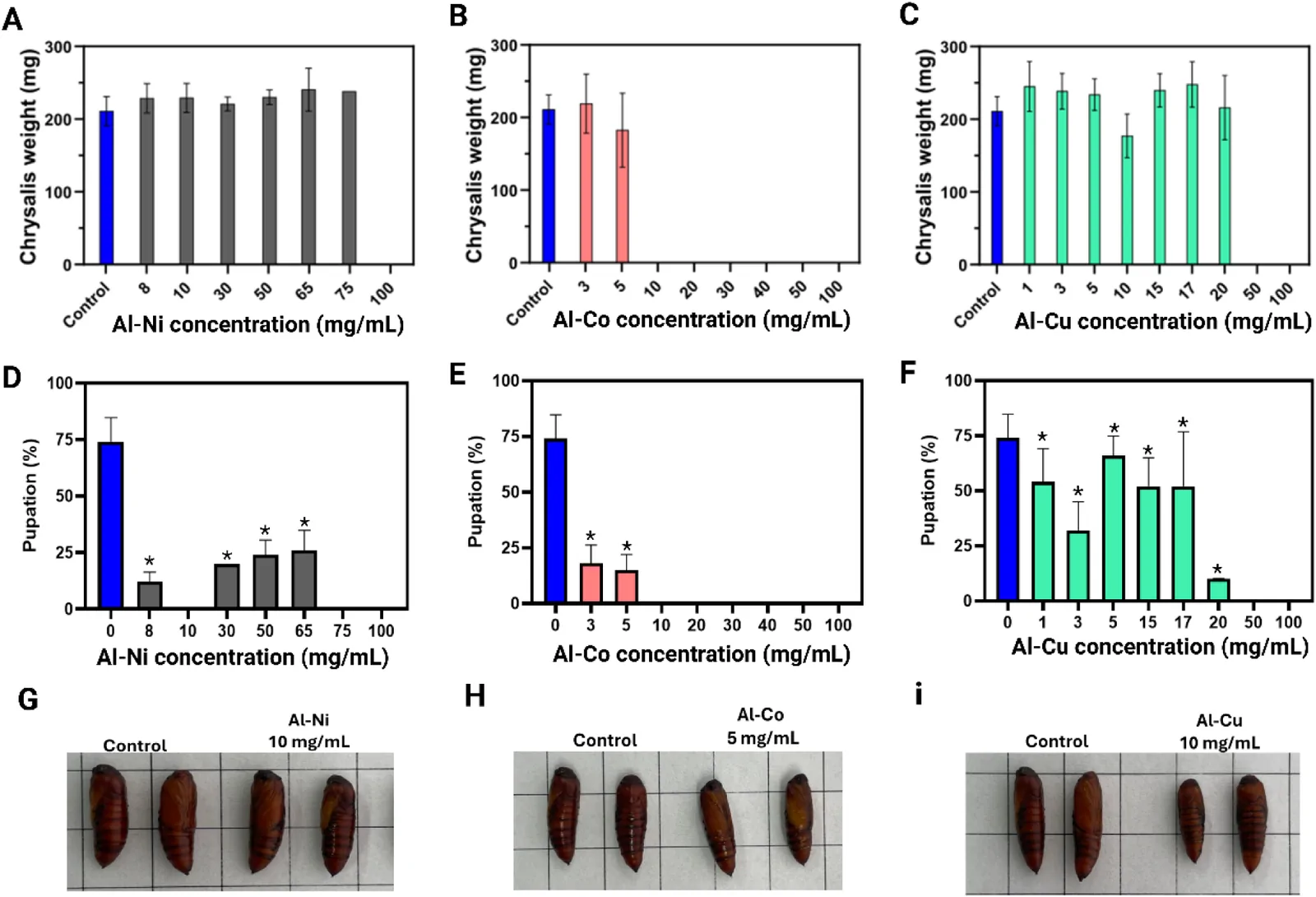
Effects of different compositions and concentrations of layered double hydroxide (LDH) nanoparticles on the larval development of *S. frugiperda*. Mean pupal weight after exposure to varying concentrations of Al–Ni, Al–Co, and Al–Cu, respectively. (D–F) Pupation percentage obtained for each treatment relative to the control, indicating the impact of nanoparticles on the metamorphosis process. (G–I) Morphological appearance of pupae from the control group and from larvae treated with 10 mg mL^–1^ of Al–Ni, 5 mg mL^–1^ of Al–Co, and Al–Cu. Morphology of surviving larvae after treatment with 10 mg mL^–1^ of each LDH type compared to the control (The photographs were taken on a reference grid composed of approximately 1 cm, allowing visualization of the reduction in body size and the delay in larval development). Results are expressed as mean ± standard deviation (*n* = 100). Differences among treatments were analyzed independently at each assessment time point using one-way ANOVA followed multiple comparison test (*p* < 0.05). Asterisks (*) indicate significant differences compared to the control group. Photographs were taken by André Lopes Ferreira.

Al–Co exposure resulted in a distinct response. Pupal weight ranged from 220 to 180 mg at 3 and 5 mg mL^–1^, without difference from the control. Remarkably, the pupation rate dropped sharply, decreasing from approximately 75% in control to below 20% at concentrations higher than 5 mg mL^–1^. Above 10 mg mL^–1^, pupae were virtually absent. These results indicate a high sensitivity of *S. frugiperda* to nanoparticles containing cobalt, suggesting that small variations in concentration are sufficient to affect physiological processes critical for metamorphosis.

The copper-containing formulation (Al–Cu) produced a gradual response. Although the mean pupal weight remained stable, ranging from 180 to 245 mg at concentrations between 1 and 100 mg mL^–1^, the pupation rate exhibited a more gradual dose-dependent response. A progressive reduction was observed, from 75% in the control to approximately 66% at 5 mg mL^–1^, with values dropping below 10% only above 20 mg mL^–1^. This difference in response suggests that, although copper is known to be a potentially more toxic metal, its release dynamics within the LDH matrix may modulate ionic bioavailability and delay deleterious effects on the organism.^32^

Taken together, the results indicate that metallic LDHs significantly impair metamorphosis in *S. frugiperda*, predominantly by reducing pupation success even when the pupae that do form maintain normal weight. Because successful pupation is essential for adult emergence and reproduction, such disruptions can dramatically reduce population viability. The distinct toxicity profiles observed among the three LDH compositions highlight the importance of metal identity, ionic solubility, and release kinetics in shaping biological outcomes. Overall, the data reinforces that LDH nanoparticles can meaningfully disrupt key developmental transitions in *S. frugiperda*, supporting their potential as innovative components in pest-management strategies while underscoring the need to evaluate environmental selectivity and effects on nontarget species.

The high development costs and limited durability of classical pest management strategies, such as chemical control and Bt crops, highlight the need for innovative approaches with multiple modes of action. Such strategies ideally should offer greater efficacy and longer-lasting performance, primarily by slowing the evolution of resistance, which directly influences the adoption and sustainability of control measures. In addition, it is increasingly important to reconsider the concept of “efficacy,” as the nonlethal effects of control tactics can significantly influence pest population dynamics and their impact on ecosystem services. In this context, our findings show that layered double hydroxide (LDH) nanoparticles containing transition metals exert pronounced toxic effects on *S. frugiperda* larvae. This species is a major agricultural pest with a wide host range, feeding on more than three hundred plant species, including economically important crops such as maize, rice, wheat, and sorghum. Without effective control measures, *S. frugiperda* infestations can severely reduce crop productivity, damage entire production areas, and lead to yield losses of up to 73%. Toxicity was dependent on nanoparticle composition, with Al–Cu LDH showing the highest potency (LD_50_: 19.1–37.3 mg mL^–1^), surpassing both Al–Ni and Al–Co formulations. Beyond acute mortality, important sublethal effects were observed, including reductions in larval and pupal weight, extended developmental time, and decreased pupation rates effects that intensified with increasing concentrations.

The biological activity observed in the present study also highlights the importance of evaluating potential effects on nontarget organisms. While LDHs have frequently been proposed as safe and biocompatible delivery systems for agricultural applications, the toxicity observed against *S. frugiperda* indicates that transition-metal-containing LDHs may not be biologically inert under all exposure conditions. Consequently, potential impacts on pollinators, natural enemies, parasitoids, soil invertebrates, and aquatic organisms should be carefully assessed. Comprehensive ecotoxicological studies will therefore be essential to determine the environmental safety profile of these materials and support their responsible development for agricultural applications.

This study has several limitations that should be considered when interpreting the results. First, although the observed effects are consistent with known metal-associated toxicity pathways, the underlying mechanisms were not directly investigated. In particular, parameters such as metal ion release, oxidative stress, or tissue-level alterations were not measured, and therefore mechanistic interpretations remain inferential. Furthermore, future experiments involving equivalent concentrations of soluble metal salts as controls (Ni^2+^, Co^2+^, and Cu^2+^), dissolution studies, and comparisons with metal-free LDHs would be important, as they would provide valuable mechanistic information. Such studies should include a direct assessment of nanoparticle internalization by cells, metal ion release kinetics, and oxidative stress biomarkers such as reactive oxygen species (ROS) production, lipid peroxidation, antioxidant enzyme activity, and DNA damage. Together, these approaches would allow for a more precise distinction between effects stemming from the lamellar structure of LDHs and those associated with metal ion release, contributing to a more comprehensive understanding of the mechanisms underlying the observed toxicity.

Second, the environmental relevance of these findings remains to be established. The potential impact of LDH nanoparticles on nontarget organisms and broader ecological systems was not assessed and requires dedicated evaluation. Third, the exposure model used in this study, based on nanoparticle incorporation into an artificial diet, does not fully replicate environmental exposure scenarios. As such, the extent to which these results translate to field conditions should be interpreted with caution. Future studies integrating environmentally relevant exposure routes and ecological assessments will be important to further evaluate the applicability of these materials.

Collectively, our findings demonstrate that metallic LDHs exert both acute and sublethal effects on *S. frugiperda*, decreasing survival, impairing development, and disrupting metamorphosis. These outcomes indicate that transition-metal-containing LDH formulations cannot be considered biologically inert under the conditions tested, exhibiting significant acute and sublethal effects on *S. frugiperda*. However, the relative contributions of the LDH matrix, metal composition, and metal-ion release remain to be determined. This discovery advances the current understanding of LDH–insect interactions and introduces a new conceptual dimension in which LDHs can be viewed as dual-function platforms, combining delivery capacity with inherent biological activity. From an applied perspective, our results position LDHs as candidates for studies on pest-control technologies, leveraging tunable metal composition to enhance potency and specificity. At the same time, the intrinsic activity we report underscores the need to evaluate selectivity, environmental fate, and potential impacts on nontarget and beneficial species before LDHs can be responsibly advanced toward field deployment.
